# Short-chain fatty acid-butyric acid ameliorates granulosa cells inflammation through regulating METTL3-mediated N6-methyladenosine modification of FOSL2 in polycystic ovarian syndrome

**DOI:** 10.1186/s13148-023-01487-9

**Published:** 2023-05-13

**Authors:** Kailu Liu, Xi He, Jingyu Huang, Simin Yu, Meiting Cui, Mengya Gao, Li Liu, Yu Qian, Ying Xie, Miao Hui, Yanli Hong, Xiaowei Nie

**Affiliations:** 1 Department of Reproductive Medicine, Jiangsu Province Hospital of Chinese Medicine, Affiliated Hospital of Nanjing University of Chinese Medicine, Nanjing, 210029 China; 2 Department of Human Anatomy and Histoembryology, Nanjing University of Chinese Medicine, Nanjing, 210023, China

**Keywords:** PCOS, Gut microbiome, Butyric acid, Inflammation, m6A modification

## Abstract

Polycystic ovary syndrome (PCOS) is an endocrine and metabolic disorder characterized by chronic low-grade inflammation. Previous studies have demonstrated that the gut microbiome can affect the host tissue cells’ mRNA N6-methyladenosine (m6A) modifications. This study aimed to understand the role of intestinal flora in ovarian cells inflammation by regulating mRNA m6A modification particularly the inflammatory state in PCOS. The gut microbiome composition of PCOS and Control groups was analyzed by 16S rRNA sequencing, and the short chain fatty acids were detected in patients’ serum by mass spectrometry methods. The level of butyric acid was found to be decreased in the serum of the obese PCOS group (FAT) compared to other groups, and this was correlated with increased Streptococcaceae and decreased Rikenellaceae based on the Spearman’s rank test. Additionally, we identified FOSL2 as a potential METTL3 target using RNA-seq and MeRIP-seq methodologies. Cellular experiments demonstrated that the addition of butyric acid led to a decrease in FOSL2 m6A methylation levels and mRNA expression by suppressing the expression of METTL3, an m6A methyltransferase. Additionally, NLRP3 protein expression and the expression of inflammatory cytokines (IL-6 and TNF-α) were downregulated in KGN cells. Butyric acid supplementation in obese PCOS mice improved ovarian function and decreased the expression of local inflammatory factors in the ovary. Taken together, the correlation between the gut microbiome and PCOS may unveil crucial mechanisms for the role of specific gut microbiota in the pathogenesis of PCOS. Furthermore, butyric acid may present new prospects for future PCOS treatments.

**Supplementary Information:**

The online version contains supplementary material available at 10.1186/s13148-023-01487-9.

## Introduction

Polycystic ovary syndrome (PCOS) is a common endocrine and metabolic disease in women. It is mainly characterized by hyperandrogenism, polycystic ovary and ovulation disorders [1]. Recent studies have shown that PCOS patients are in a state of chronic low-grade inflammation [2, 3]. This chronic inflammatory state is aggravated by obesity. Moreover, chronic low-grade inflammation promotes the development of ovarian and metabolic dysfunction in PCOS. A higher concentration of many cytokines and chemokines, such as interleukin-6 (IL-6) and tumor necrosis factor-α (TNF-α), have been found to play a role in the development of ovarian and metabolic dysfunction in PCOS [4]. In addition, a higher concentration of these cytokines and chemokines contributes to the development of reproductive abnormalities such as follicle development perturbation [5]. Therefore, understanding the regulatory mechanisms of inflammation is necessary to find pharmacological agents that can treat PCOS.

N6-methyladenosine (m6A) is one of the most common forms of RNA modifications in mammals [6]. The modification of m6A in RNA is involved in many important biological functions, such as RNA metabolism [7], spermatogenesis [8], and embryo development [9]. Researchers have also discovered that m6A modification affects cell inflammation by regulating inflammation-related genes [10]. For example, RNA methyltransferase-METTL3 regulates the NF-κB inflammatory pathway by upregulating the m6A modification level of TRAF6 [11]. In addition, METTL3/METTL14 increases Transforming Growth Factor Beta-1 (TGF-β1) 5'UTR mRNA m6A modification by inducing lipopolysaccharide (LPS), resulting in hepatocyte fibrosis [12]. The gut microbiome affects many aspects of human health and disease, resulting in various host reactions, including significant genetic changes. It has been found that microorganisms can also affect the m6A modification level of mRNA in the host tissues [13, 14]. The change in intestinal flora diversity is strongly correlated with PCOS [15, 16]. Thus, the gut microbiome is involved in the pathogenesis of PCOS via multiple pathways [17]. However, it is unclear whether the intestinal flora affects the inflammation of ovarian cells by mRNA m6A modification, particularly in the PCOS state.

In this study, we conducted an analysis of the gut microbiome population distribution in individuals with PCOS and healthy Controls. We also measured the serum concentration of short-chain fatty acids (SCFAs) in PCOS and control patients, with a particular focus on butyric acid. Further, we established a cellular model for overexpression of METTL3 and performed Methylated RNA immunoprecipitation sequencing (Me-RIP) to investigate the effect of butyrate acid on KGN cells epigenetic modifications, particularly m6A modification, under inflammatory conditions. This study improves the understanding of PCOS pathogenesis and ideas for developing novel therapies.

## Results

### Clinical characteristics of the participants

A total of 18 patients, including 6 male infertility patients or patients receiving in vitro fertilization (IVF) treatment for tubal factors in the control group (control group) and 12 patients with PCOS, were recruited to participate in this study. The patients were divided into two groups; the mere obese group (FAT) and the mere hyperandrogenism group (HA) according to body mass index (BMI) and serum androgen levels. The demographic and clinical characteristics of the study participants, including age, body mass index (BMI), anti-Müllerian hormone (AMH), follicle-stimulating hormone (FSH), luteinizing hormone (LH), and testosterone (T), are presented in Table 1. Age was comparable among the three groups. However, patients with polycystic ovary syndrome (PCOS) exhibited significantly higher levels of serum AMH, LH, and LH/FSH ratio compared to the control group. No significant differences in T levels were observed between the FAT and control groups. Our findings suggest that PCOS patients have elevated plasma levels of pro-inflammatory cytokines when compared to healthy individuals. Furthermore, within the PCOS subgroups, obese patients in the FAT group showed higher levels of IL-6 (93.53 ± 12.96 pg/ml vs. 49.17 ± 9.49 pg/ml) and TNF-α (195.20 ± 27.02 pg/ml vs. 124.01 ± 11.17 pg/ml) compared to the HA group, which is consistent with previous studies [18, 19]. These results indicate a more robust inflammatory response in obese PCOS patients.

**Table 1:** Comparison of clinical features between the three groups

	Control (*n* = 6)	FAT (*n* = 6)	HA (*n* = 6)
Age (year)	27.50 ± 2.59	27.50 ± 2.88	28.83 ± 3.76
BMI (kg/m^2^)	20.26 ± 2.38	27.16 ± 2.20^A^	24.42 ± 3.56
AMH (ng/ml)	2.77 ± 0.59	5.66 ± 0.64^A^	5.47 ± 1.05^B^
FSH (U/L)	7.26 ± 2.05	5.97 ± 1.42^A^	5.74 ± 1.04^A^
LH (U/L)	2.52 ± 1.27	4.34 ± 1.93	11.46 ± 2.39
T (ng/dL)	44.91 ± 9.36	45.25 ± 20.80	104.10 ± 26.61^B,C^
E2 (pg/ml)	49.17 ± 29.11	50.67 ± 27.15	40.67 ± 7.55
TNF-α (pg/ml)	78.48 ± 12.93	203.01 ± 29.55^A^	119.50 ± 4.48^B,C^
IL-6 (pg/ml)	21.42 ± 6.87	93.65 ± 8.65^A^	49.18 ± 5.82^B,C^

^A^*P* < 0.05 for control versus FAT group

^B^*P* < 0.05 for control versus HA group

^C^*P* < 0.05 for FAT versus HA group

### Obesity affects the microbial diversity and composition of PCOS patients

**Fig. 1:**
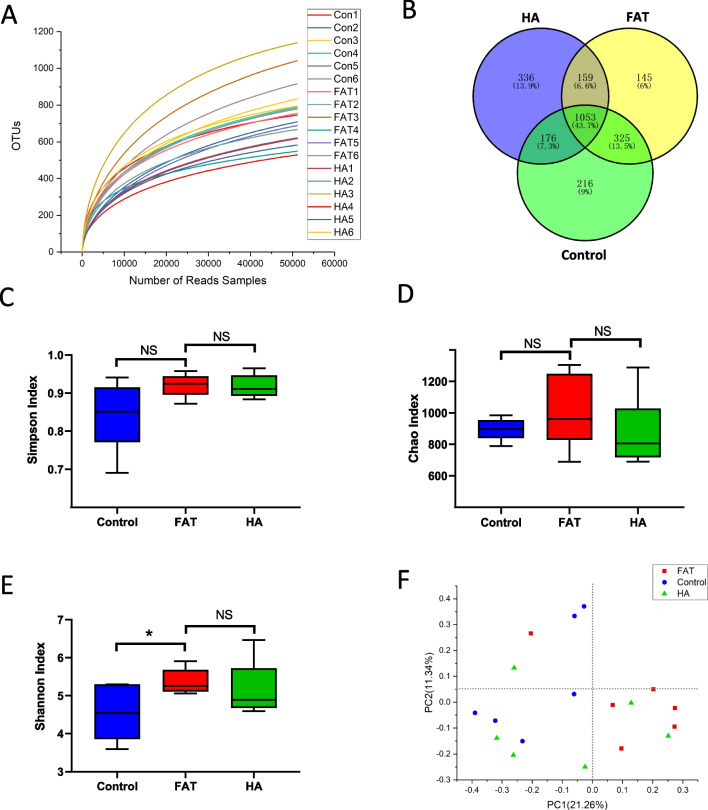
Diversity analysis of gut microbiota among three groups. **A** Shannon–Wiener curves, showing that the amount of sequencing data is large enough to reflect the vast majority of microbial information in the samples. **B** Venn diagram, showing the number of common and unique OTUs, and the similarity and overlap of OTUs among groups. **C** Simpson index of OTUs, Chao indexes of OTUs (**D**), and Shannon index (**E**) of OTUs in three groups. *P < 0.05. **F** Principal co-ordinates analysis (PCoA analysis). PC1 and PC2 represent the two suspected influencing factors of microbial composition migration. Percentage represents the contribution of principal coordinate components to sample composition differences. The greater the proximity between two sample points, the higher the degree of similarity in terms of species composition between the two samples

We collected the feces of three groups of patients and analyzed the distribution of microflora using 16S sequencing to explore the effect of obesity on the intestinal flora of PCOS patients. The 16 s sequencing data showed that the Operational Taxonomic Unit (OTU) numbers of the three groups were similar. The results also demonstrated that 1053 OTU were co-expressed in the three groups, as shown in Fig. 1A, B. Measures of microbial diversity (α-diversity) revealed that obesity did not affect the Simpson and Chao1 species (OTU) richness in the FAT group (Fig. 1C, D). However, Shannon’s diversity index was lower in the normal group (Fig. 1E). The β-diversity was calculated and ordinated using PCoA to compare the microbial composition between the three groups. Samples from the FAT group were differentiated from the HA and Control groups, as shown in Fig. 1F. These analyses revealed that microbial composition was significantly different between the three groups.

### The abnormal gut microbiota in obese PCOS reduces butyrate acid

**Fig. 2:**
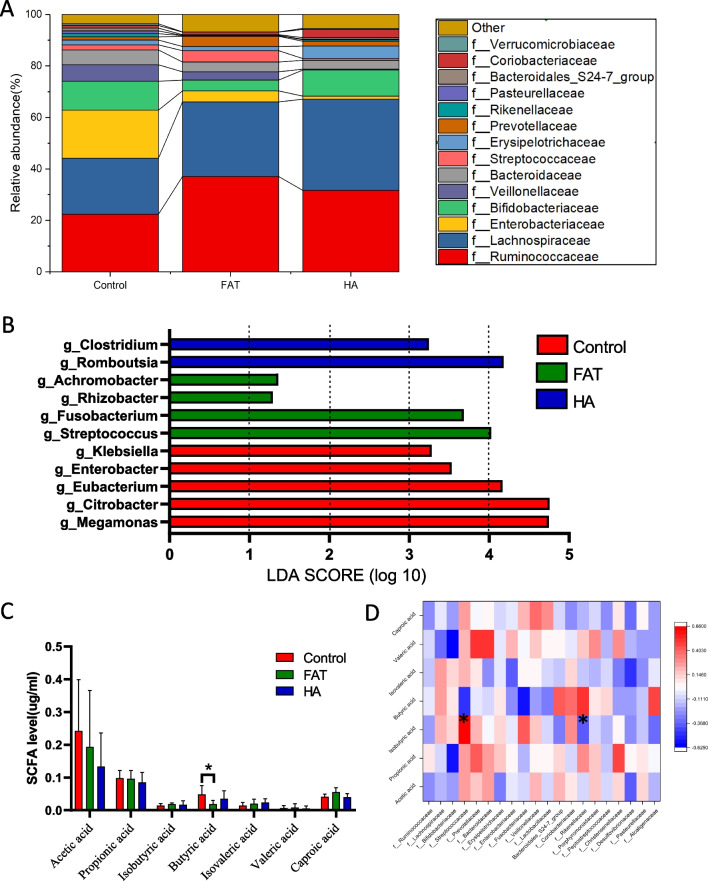
Composition and Linear discriminant analysis of gut microbiota among three groups. **A** Composition of species at family level among three groups. **B** Linear discriminant analysis (LDA) discriminant histogram of gut microbiota in three groups. Identification of the bacterial taxa with statistically significant difference between groups using LEfSe software and LDA. Taxa enriched in Control, FAT-PCOS, and HA-PCOS group are colored by red, green, and blue respectively (LDA > 2.0 and *P* < 0.05), the relative abundance of thesebiomarkers are shown in the histogram (mean and standard deviation values are plotted) under the corresponding cladogram. **C** The content of short chain fatty acids in serum of three groups of patients. *P < 0.05. **D** Spearman correlation heat map between clinical parameters and flora. The color of spots represents the Spearman correlation R-value between each bacterial taxa and clinical parameters. *P < 0.05

In this study, we analyzed the distribution of intestinal flora in the three groups of patients and found that the main types of flora in the three groups were similar at the family level. However, there were differences in their respective dominant flora (Additional file 1: Fig. S2A). For instance, Enterobacteriaceae and Bifidobacteriaceae were the predominant groups of bacteria in the intestinal microbiota in the normal group. On the other hand, Ruminococcacea and Lachnospiraceae were the dominant bacterial groups in the FAT group. The abundance of Prevotellaceae family is higher in the PCOS fecal samples compared to the control group, as shown in Fig. 2A. The dominant flora of each group at the genus level was analyzed using pairwise LEfSe analysis (LDA score > 2.0 and *P* < 0.05). Streptococcus, Fusobacterium, Rhizobacter, and Achromobacter were the representative flora in the FAT group, and Clostridium and Romboutsia were abundant in the HA group (Fig. 2B). The content of SCFAs in the serum of patients in each group was analyzed. The results showed that the level of butyric acid in the FAT group had decreased. However, the level of butyric acid was not significantly different between the FAT group and the HA and control groups (Fig. 2C). A combined Spearman’s rank test of the flora and SCFAs found that the increased level of Streptococcaceae and decreased Rikenellaceae affect the butyric acid in PCOS patients (Fig. 2D).

### Butyric acid improves LPS-induced apoptosis and oxidative stress

**Fig. 3:**
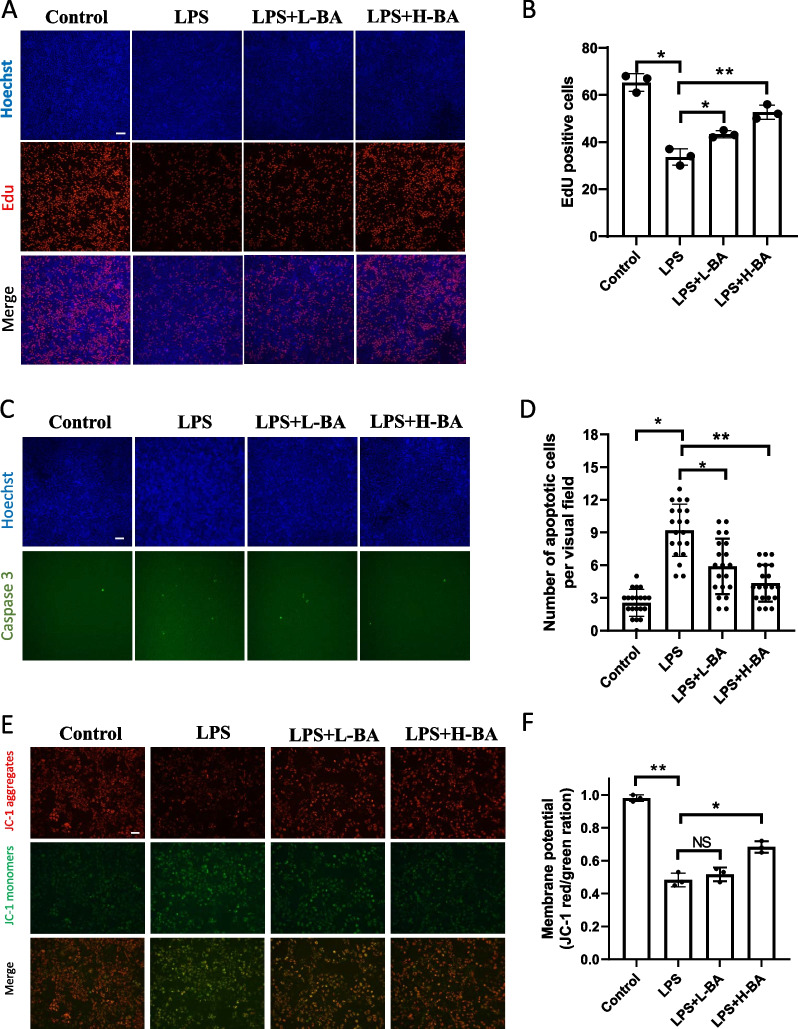
Butyric acid can improve the metabolism and inflammatory response of granulosa cells in inflammatory state. **A**, **B** Edu staining was used to detect KGN cells proliferation treated with LPS and low-dose(L-BA) and high-dose (H-BA) butyric acid. Scale bar: 100um, *P < 0.05, **P < 0.01. **C**, **D** Caspase3 activity kit was used to detect apoptotic KGN cells treated with LPS and low-dose and high-dose butyric acid for 48 h. Relative caspase3 activity was calculated by average positive cell ratio (The results were shown as means ± SD) (*n* = 3). Scale bar: 100um, *P < 0.05, **P < 0.01. **E**, **F** Mitochondrial membrane potential was detected by JC-1 staining. Red fluorescence indicates high mitochondrial membrane potential, while green fluorescence indicates low mitochondrial membrane potential. The higher the red/green ratio, the fewer apoptotic cells. (The results were shown as means ± SD) (*n* = 3). Scale bar: 100um, *P < 0.05, **P < 0.01

**Fig. 4:**
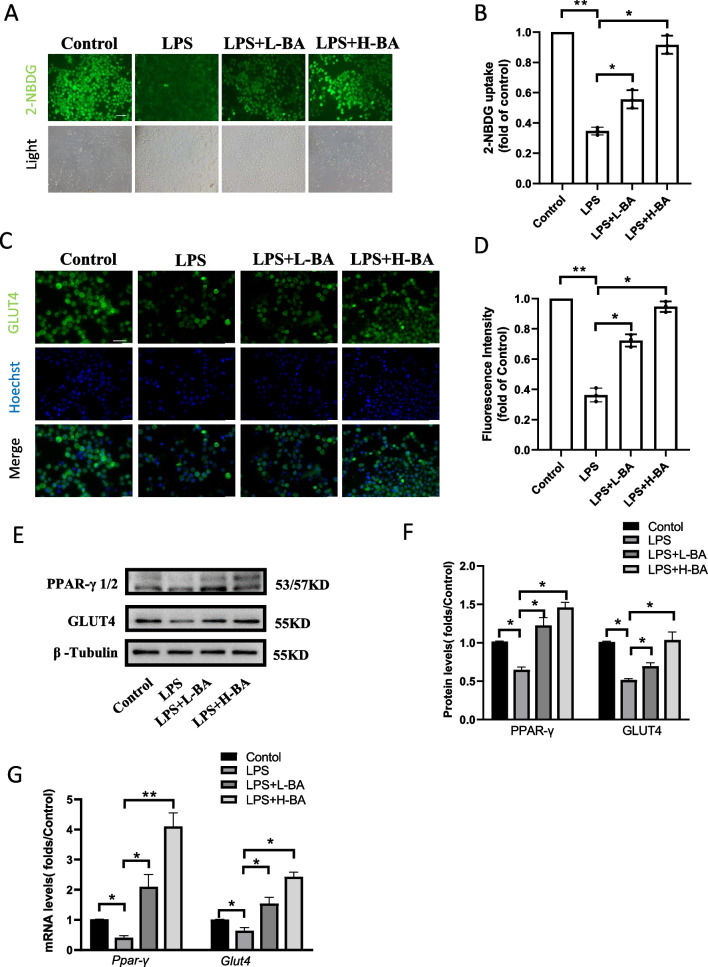
Butyric acid improves glucose transport in inflammatory KGN cells. **A**, **B** Glucose uptake of KGN cells was detected by 2-NBDG staining. Fluorescence intensity of 2-NBDG (green color) was determined by Image J software. Each value in the graph represents the relative units (fluorescence/cell numbers). Scale bar, 100 μm. *P < 0.05, **P < 0.01. **C**, **D** The distribution of GLUT4 in KGN cells was showed by immunofluorescence. Scale bar, 20 μm. *P < 0.05, **P < 0.01. **E**, **F** The protein expression of PPAR-γ1/2 and GLUT4 in the KGN cells after treated with butyric acid. *P < 0.05, *n* = 3. **G** The mRNA expression of PPAR-γ and GLUT4 in KGN cells after treated with butyric acid.. *P < 0.05, ** < 0.01, *n* = 3

LPS was used to stimulate human ovarian granulosa tumor (KGN) cells to construct the inflammatory state of PCOS patients. The KGN cells were treated with LPS for 12 h. After LPS treatment, the cells were treated with butyric acid of different concentrations for 12 h. The effect of butyric acid on inflammatory KGN cells was observed and recorded. Subsequently, KGN cells were divided into a low-dose group (L-BA) and a high-dose group (H-BA) according to the dosage of butyric acid. The Edu method was used to detect the level of cell proliferation. The results showed that the proliferation level of KGN decreased after LPS treatment and recovered after butyric acid treatment (Fig. 3A, B). Caspase3 detection showed that the apoptotic cells increased significantly after adding LPS but decreased after adding butyric acid (Fig. 3C, D). Since several studies have shown that butyric acid can improve mitochondrial function, JC-1 was used to detect mitochondrial membrane potential. The fluorescence results showed that butyric acid could improve mitochondrial membrane potential, as shown in Fig. 3E, F. Studies have reported that butyric acid can activate PPAR-γ, which increases the expression of GLUT4, improving the glucose uptake ability of cells. Therefore, we used the 2-NBDG fluorescence tracer method to detect the glucose uptake capacity of KGN cells. The results showed that LPS decreased and butyric acid improved the glucose uptake capacity of KGN cells (Fig. 4A, B). The expression and localization of GLUT4 in KGN cells in each group were detected using an immunofluorescence assay. The immunofluorescence staining showed that the expression of GLUT4 protein was higher in butyric acid-treated groups than LPS treated groups (Fig. 4C, D). In addition, QRT-PCR and western blot showed that PPAR-γ, GLUT4 mRNA, and protein were more highly expressed in butyric acid-treated KGN cells than in LPS-treated KGN cells (Fig. 4E–G). The results indicated that butyric acid could improve the glucose metabolism of KGN cells in an inflammatory state.

### Butyric acid inhibits inflammatory cytokine expression by inhibiting the expression of METTL3

**Fig. 5:**
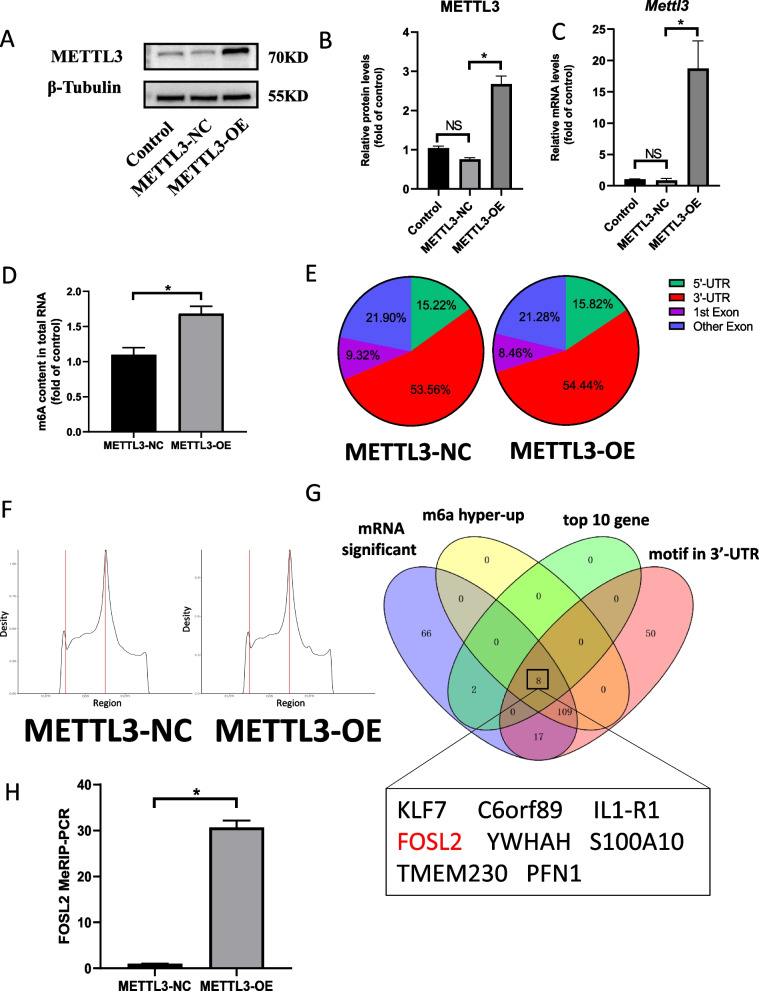
Sequencing analysis of KGN cells m6A. **A**, **B** The overexpression METTL3 plasmid was constructed. 24 h after it was transferred into KGN cells, the protein expression level of METTL3 was detected. The results showed that the expression of METTL3 in the overexpression plasmid group (OE) was significantly higher than that in the blank plasmid group (NC). Bars represent means ± SD, *n* = 3. *P < 0.05. **C** MRNA level of METTL3 increased significantly in the overexpression group. Bars represent means ± SD, *n* = 3. *P < 0.05. **D** Distribution of m6A fragments enriched by two groups of KGN cells in RNA, most of which are located in 3’UTR region. **E** Quantification analysis of m6A modification in the KGN cells of the METTL3-NC and METTL3-OE group. The m6A content in total RNA of KGN cells were detected by colorimetric assay. Bars represent means ± SD, *n* = 3. *P < 0.05 vs the control. **F** Metagene profiles of enrichment of m6A peaks across mRNA transcriptome of the METTL3-NC and METTL3-OE group. **G** Veen diagram shows that through enrichment analysis, eight potential targets are selected according to mRNA expression level, m6A modification level and motif region, and FOSL2 is determined as a target for subsequent research in combination with relevant literature. **H** The expression amount of FOSL2 mRNA motif1 in KGN cells of the two groups. qpcr results showed that when METTL3 was overexpressed in KGN, the expression of FOSL2 m6A modified motif1 increased. Bars represent means ± SD, *n* = 3. *P < 0.05

**Fig. 6:**
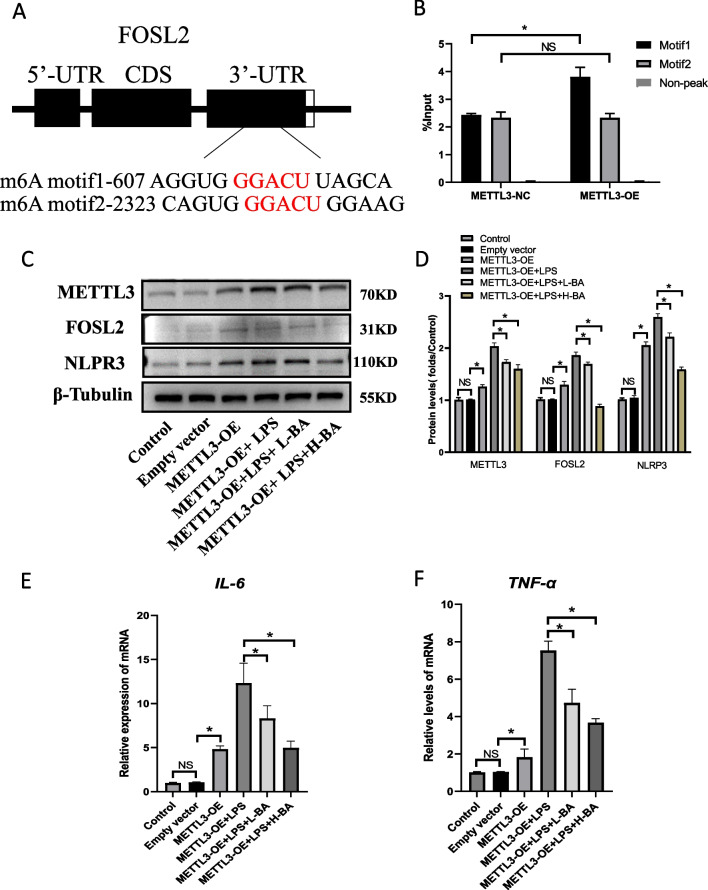
Butyric acid inhibits inflammation through METTL3/FOSL2/NLRP3 pathway. **A**, **B** qRT-PCR analysis of m6A motif1 and motif2 and non-peak region in the 3’-UTR of the FOSL2 transcript. Bars represent means ± SD, *n* = 3. *P < 0.05 for the differences between the indicated groups. **C**, **D** On the basis of overexpression of METTL3, we added LPS pretreatment, and then added low concentration butyric acid and high concentration butyric acid to detect the protein expression levels of METTL3, FOSL2 and NLRP3. Bars represent means ± SD, *n* = 3. *P < 0.05, **P < 0.01. **E**, **F** The mRNA expression level of IL-6 and TNF-α in several groups of KGN cells. Bars represent means ± SD, *n* = 3. *P < 0.05

A METTL3 overexpression plasmid was constructed and transferred into KGN cells, and we used WB and QPCR to detect the overexpression efficiency of METTL3 and found that METTL3 mRNA and protein were highly expressed in the METTL3- overexpression (METTL3-OE) group than in the METTL3-normal control (METTL3-NC) group (Fig. 5A–C). The m6A ratio of the METTL3-NC and METTL3-OE groups was analyzed. An m6A kit was used to detect the content of m6A in the total RNA of KGN cells in two groups. The results showed that the level of m6A modification in the METTL3-OE group was significantly higher than in the METTL3-NC group (Fig. 5D). The MERIP sequencing results showed that the enriched sequence was AAGGACC (Additional file 1: Fig. S2B). The sequence met the requirements of GGACU of the m6A modified site. Moreover, the modification site of m6A is mainly located in the 3'UTR region, as shown in Fig. 5E, F. Analyzing the MERIP sequencing results showed that FOSL2 might be a potential METTL3 regulatory target (Fig. 5G). The m6A content of FOSL2 was detected using the MERIP kit. The findings demonstrated that the m6A-modified mRNA expression of FOSL2 was increased in the METTL3 overexpression group (Fig. 5H). Two m6A modified sites were found when searching the location of GGACU in the 3’UTR section of FOSL2 in the database. There were significant differences between the two groups in motif 1, suggesting that METTL3 acts on motif 1 (Fig. 6A, B). We also detected the expression of FOSL2 mRNA and protein in KGN cells overexpressing METTL3. The results showed that the expression of FOSL2 in KGN cells overexpressing METTL3 was increased. The increase in FOSL2 was more obvious after adding LPS (Fig. 6C, D). The protein expression of NLRP3 was also detected. The findings showed that the expression trend of NLRP3 was consistent with FOSL2, as shown in Fig. 6C, D. Since studies have shown that FOSL2 can promote the expression of inflammatory factors, inflammatory factors IL-6 and TNF-α were tested. It was found that the expression trend of these two inflammatory factors was consistent with FOSL2 (Fig. 6E, F). To verify the regulatory role of FOSL2 on IL-6 and TNF-a, we used siRNA to knockdown FOSL2 in KGN cells and measured the expression of IL-6 and TNF-α (Additional file 1: Fig. S2C, S2D, S2E). We found that the protein levels of IL-6 and TNF-α were decreased when FOSL2 expression was downregulated in KGN cells (Additional file 1: Fig. S2F, S2G).

### The effect of butyric acid on mice ovary

**Fig. 7:**
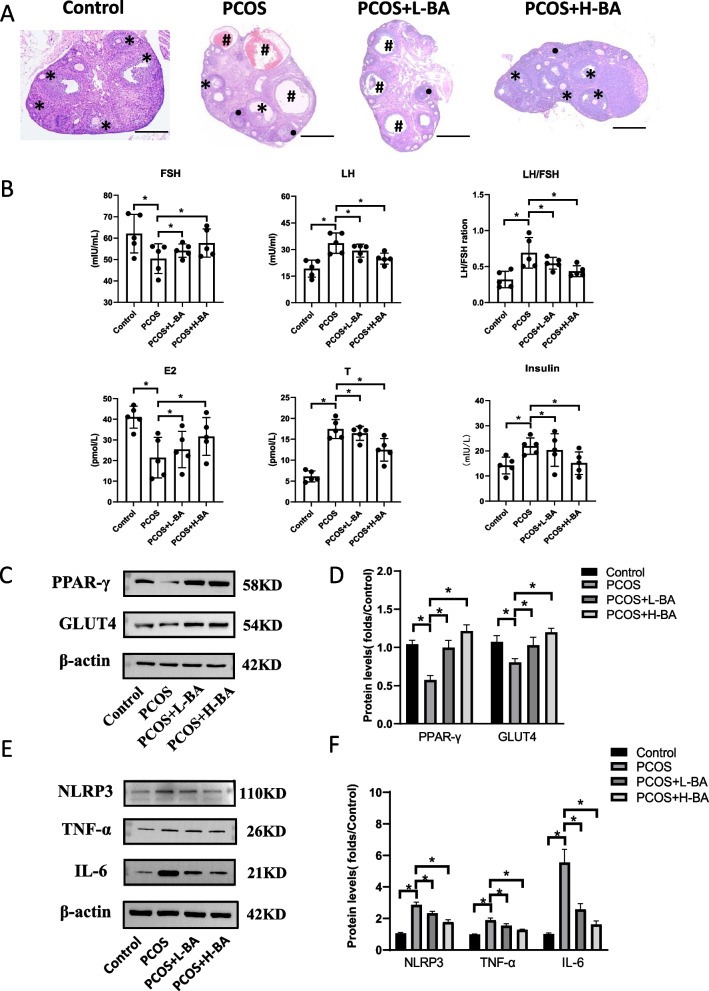
Butyric acid improves ovarian inflammation in PCOS model mice. **A** Photomicrographs of representative ovarian cross section from four groups: Control group, PCOS group, PCOS + Low BA group, and PCOS + High BA group. *: developing follicles; ●: corpus luteum; #: cystic follicles. Bar = 500 μm. **B** Serum hormone level of mice in each group. Bars represent means ± SD, *n* = 5. *P < 0.05. **C**, **D** PPAR-γ and GLUT4 in ovarian tissue of mice in each group protein expression level. Bars represent means ± SD, *n* = 5. *P < 0.05. **E**, **F** NLRP3, TNF-α and IL-6 in ovarian tissue of mice in each group protein expression level. Bars represent means ± SD, *n* = 5. *P < 0.05

A mice model of obese PCOS was created to investigate the impact of butyric acid on ovary function. The mice were divided into four groups, namely the control group, the obese PCOS model group, the low-dose butyric acid group, and the high-dose butyric acid group. No significant difference in mice quality was observed before the experiment commenced, and no weight loss was observed after one week of intraperitoneal injection of butyric acid (Additional file 1: Fig. S2H). The ovarian histomorphology of mice in each group was different. The ovaries of mice in the control group were bright red, without expanding follicles. Histomorphological analysis of the ovaries revealed significant differences among the groups, with the ovaries of the obese PCOS model mice showing multiple follicles of varying sizes, while the control group exhibited bright red ovaries without expanding follicles. The low-dose butyric acid treatment did not significantly alter ovarian morphology, but the high-dose butyric acid treatment reduced the number of ovarian follicles, as shown in Fig. 7A. ELISA was used to detect the serum concentration of FSH, LH, T, E2, and insulin of mice in each group. It was observed that low doses of butyric acid could improve the level of hormones in PCOS mice. The level of related hormones changed significantly after taking a high concentration of butyric acid (Fig. 7B). The expression levels of PPAR-γ and GLUT4 were also detected in the ovaries of mice in each group. The protein expression levels of PPAR-γ and GLUT4 decreased in PCOS model mice and increased after taking butyric acid (Fig. 7C, D). The expression of NLRP3, IL-6 and TNF-α in the ovaries of mice decreased after taking butyric acid (Fig. 7E, F).

## Discussion

Polycystic ovary syndrome is an extremely complex metabolic and endocrine disease whose molecular mechanisms of pathogenesis remain unclear. However, it is widely accepted that the complex interplay between genetic predisposition, hormonal disturbances, and environmental factors contribute to the development of PCOS [20–22]. Patients with PCOS often present with obesity, insulin resistance (IR), cardiovascular disease, and other long-term metabolic syndromes [23, 24]. PCOS is a leading cause of infertility in women, and its prevalence has been increasing steadily over the past few decades [25]. Obesity is the most common complication of PCOS. It affects about 50% of PCOS patients worldwide [26]. In addition, obesity aggravates abnormal metabolism in PCOS patients, affecting oocyte quality and endometrial reception [27, 28].

Evidence from research shows that chronic inflammation is a risk factor for PCOS, and inflammatory factors are constantly higher in PCOS patients [3]. An abnormal increase in inflammatory factors would lead to multiple negative effects for PCOS. On the one hand, abnormal expression of inflammatory factors in peripheral circulation and ovarian tissue of PCOS patients could induce immune dysfunction and ovulation disorder [29, 30]. A dysfunction in the immune system affects follicular development or ovulation [31]. On the other hand, an increase in inflammatory factors in PCOS patients changes the level of AMH, resulting in glucose and lipid metabolism disorder [32, 33]. This disorder increases the risk of IR and synthesis of androgens, destroying normal ovulation and fertilization in women. In addition, the disorder results in PCOS symptoms characterized by menstrual disorders and abnormal ovulation [34]. The inflammatory factors IL-6 and TNF-α were detected in patients in the normal and PCOS groups. The findings showed that IL-6 and TNF-α were higher in the PCOS group than in the normal group (*P* < 0.05). In the PCOS group, IL-6 and TNF-α in the FAT group were higher than in the HA group (*P* < 0.05). Our findings were consistent with previous studies that the obese PCOS patients had more significant inflammation [35, 36].

Studies on gut microbiota mainly focused on the pathogenesis of PCOS by reducing beneficial metabolites and increasing harmful substances, such as LPS and peptidoglycan. The microbiome affects the host physiology and regulates host gene expression by their metabolites, such as SCFAs, amino acids, and bile acids [37]. However, studies on the gut microbial community in PCOS were inconsistent due to differences in ethnicity, sample type, dietary habits, and environmental factors. For instance, Zeng et al. reported that Prevotellaceae was lower in PCOS patients than in healthy people, especially in patients with insulin resistance [38]. Moreover, some researchers have shown that Lactococcus and Coprococcus 2 are the characteristic gut microbiota in non-obese and obese PCOS patients, respectively [16]. Liu found that Gram-negative bacteria belonging to Bacteroides and Escherichia/Shigella were increased, and fecal bacilli, Bifidobacteria and Brucella were decreased in the intestines of PCOS patients [39]. An in-depth study of intestinal flora found that high-sugar and high-fat diets in PCOS patients result in intestinal flora disorder and damage intestinal mucosa integrity. In addition, a high-sugar and high-fat diet activates the immune system, inhibits the function of the insulin receptor, improves the serum insulin level and causes the LPS on the intestinal bacterial cell wall to enter the blood circulation. As a result, it increases the production of androgens in the ovary, interfering with the normal development of follicles and resulting in PCOS [40]. Studies have reported that the altered gut microbial community participates in the pathogenesis of PCOS through the Bile acid-intestinal FXR-insulin axis [41].

SCFAs are volatile fatty acids produced by the intestinal microbiome in the fermentation of undigested dietary fiber [42]. Zhang et al. found that the contents of acetic acid, propionic acid and butyric acid in PCOS patients were significantly lower than those in the control group [43]. The results demonstrated that butyric acid decreased more in obese PCOS patients than in the control and the hyperandrogenic-type PCOS groups. Butyric acid, the most abundant short-chain fatty acid, is the major energy source for colonocytes. In addition, it has anti-inflammatory, antioxidant and anti-cancer effects [44]. Studies have shown that butyric acid promotes the secretion of intestinal hormones, such as glucagon-like peptide and 5-hydroxytryptamine, by interacting with enterocyte surface receptor 41 (GPCR41) or GPCR43 [45]. Another study found that oral treatment of mice with sodium butyrate significantly improved insulin sensitivity in diabetic mice [46]. We confirmed that butyric acid could improve the LPS-induced apoptosis and the glucose metabolism of KGN cells through PPAR-γ/GLUT4.

The M6A modification, the most common type of RNA modification, is involved in many important physiological and pathological processes in the reproductive system [47, 48]. It also plays an important role in premature ovarian failure and PCOS [49, 50]. The increased expression of methylation enzymes METTL3 and METTL14 in patients with PCOS lead to an increase in the m6A modification level. The increased m6A modification affects the function of granulosa cells in PCOS patients by regulating FOXO3 m6A methylation levels [49]. Therefore, understanding the role of the m6A modification can provide insights into the pathophysiology of PCOS and facilitate the development of new therapeutic approaches for the condition. Interestingly, several studies have shown that changes in intestinal flora can change the epigenetics of the host. Wang et al. noted that the microbiome strongly affects the host m6A mRNA modification overexpression in the brain. The author also detected strong changes in the expression of the methyltransferase METTL3 in the brain [14]. However, the role of intestinal flora in the epigenetic modification of the ovary is yet to be reported. This finding highlights the potential therapeutic benefits of targeting the gut microbiome in the treatment of reproductive disorders. Further research is necessary to shed more light on the epigenetic modifications of the ovary and the potential therapeutic benefits of targeting the gut microbiome in the treatment of reproductive disorders.

By conducting cell and animal experiments, we discovered that butyric acid possesses the ability to repress the activity of methylase METTL3, while FOSL2, a member of the AP1 family, crucially participates in the immune response, and facilitates the expression of inflammatory factors [51]. Upon detecting the m6A modification site of FOSL2, we concluded that the METTL3-OE and METTL3-NC groups significantly differed in one of the modification sites. Our results further demonstrated that the overexpression of METTL3 led to increased expression of FOSL2 in KGN cells, whereas the addition of butyric acid effectively decreased its expression, along with the concurrent decrease in the expression of NLRP3 and inflammatory factors IL-6 and TNF-α. Studies have shown that *Fusobacterium nucleatum* reduces METTL3-mediated m6A modification by inhibitting the Hippo pathway and activating the Yes-Associated Protein (YAP) signal pathway. The Yap get into the nucleus and affected the transcription promotion of FOXD3 on METTL3, resulting in a systematic decrease in the level of m6A modification of overall mRNA in colorectal cancer cells [52]. Butyric acid could activate YAP in human intestinal smooth muscle cells [53]. However, further studies are needed to explore whether butyric acid inhibits METTL3 through the YAP pathway or influences METTL3 expression through other proteins.

**Fig. 8:**
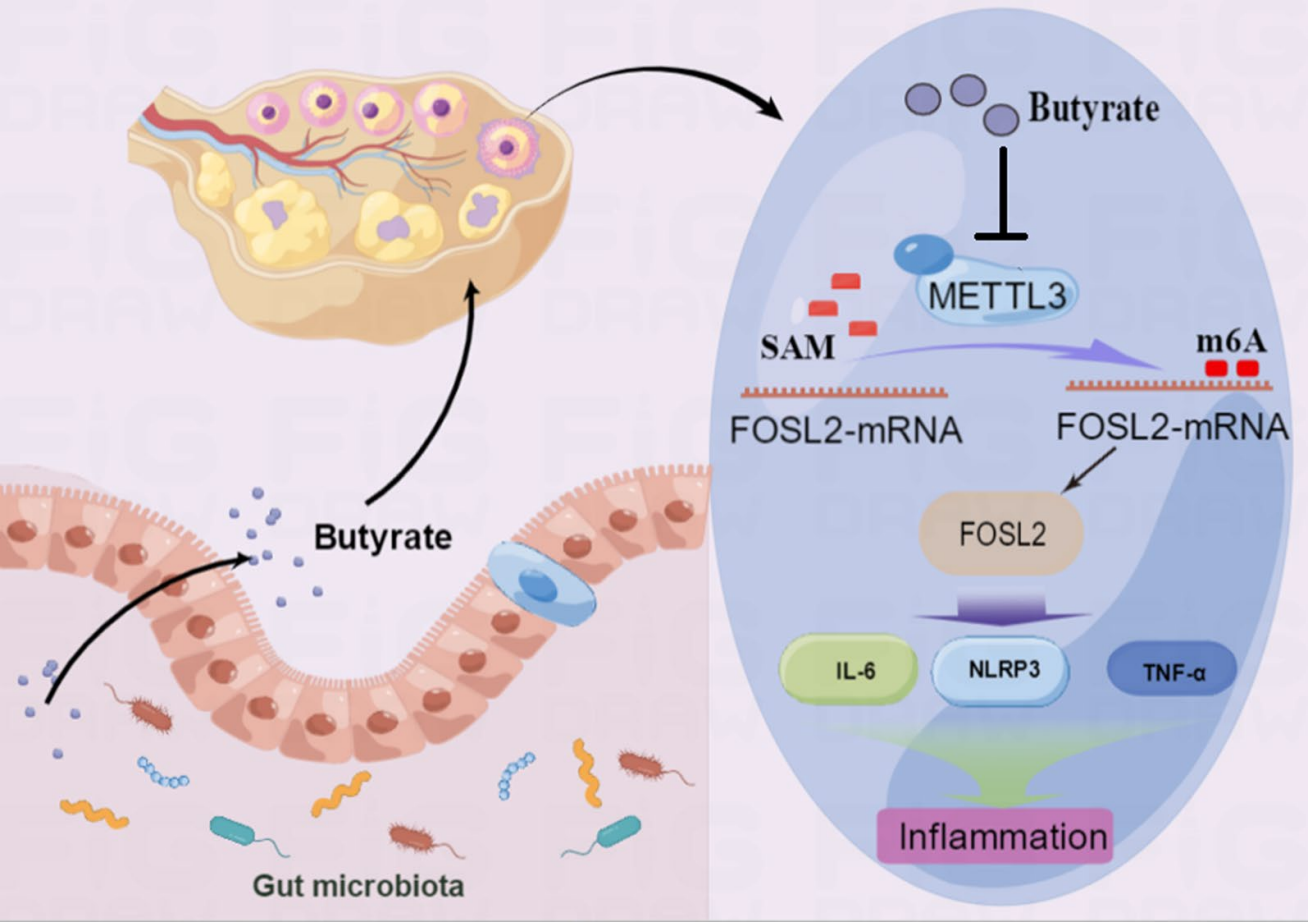
Butyric acid improves ovarian inflammation by inhibiting METTL3/FOSL2/NLRP3. Schematic representation of the effect of butyric acid on ovarian inflammation by inhibiting METTL3/FOSL2/NLRP3 pathway. The butyric acid produced by specific intestinal flora enters the blood circulation and finally reached the ovarian tissue. Under normal conditions, butyric acid could downregulate METTL3, thus reducing the m6A modification of FOSL2, finally reduced the downstream inflammatory reaction. In PCOS patients, due to the reduced of butyric acid, disorders of the intestinal flora lead to ovarian inflammation

In conclusion, this study aimed to investigate the role of gut microbiota in ovarian cells inflammation in PCOS by regulating mRNA m6A modification. The study discovered a correlation between gut microbiota composition and PCOS. Butyric acid, which was found to be decreased in PCOS patients, was identified as a potential METTL3 target. Cellular experiments showed that butyric acid supplementation led to a decrease in FOSL2 m6A methylation levels and mRNA expression by suppressing METTL3 expression (Fig. 8). Additionally, butyric acid supplementation in obese PCOS mice improved ovarian function and decreased the expression of local inflammatory factors in the ovary. These findings suggest that gut microbiota play a crucial role in the pathogenesis of PCOS and butyric acid has potential as a future treatment for PCOS.

## Materials and methods

### Clinical patients data

This study was approved by the Ethics Committee of the Affiliated Hospital of Nanjing University of Chinese Medicine (Ethics No. 2019NL-194-02). The participants signed the informed consent. The Rotterdam criteria was used to diagnose polycystic ovarian syndrome (PCOS) [54]. The exclusion criteria include: hypertension, cardiovascular disease, hyperprolactinemia, diabetes, liver or renal disease, endometriosis, ovarian tumors, ovarian insufficiency, and other conditions affecting follicular development. The standard of obese PCOS is BMI ≥ 25 kg/m [55]. In this study, the participants were not treated with Glucocorticoids, oral contraceptives, immunomodulatory drugs, insulin sensitizers, and antibiotics.

A total of 18 patients, including 6 male infertility patients or patients receiving IVF treatment for tubal factors in the control group (Control group) and 12 patients with PCOS, were recruited to participate in this study. The patients were divided into two groups; the mere obese group (FAT) and the mere hyperandrogenism group (HA) according to body mass index (BMI) and serum androgen levels. Participants were included in the HA group if their androgen levels were ≥ 70 ng/dl with a clear diagnosis of PCOS. Blood was drawn from the fasting vein 2–3 days after the onset of menstruation. Subsequently, the levels of sex hormones, including follicle-stimulating hormone (FSH), luteinizing hormone (LH), estrogen (E2), testosterone (T), AMH, IL-6, and TNF-α, were detected using chemiluminescence enzyme-linked immunosorbent assay.

### Intestinal flora analysis

To analyze the intestinal flora, 16S ribosomal ribonucleic acid (rRNA) sequencing was performed on stool samples. DNA was extracted from the stool samples of the three groups and pre-amplified and sequenced by Feng Zi Biomedical Technology (Nanjing, China). The Qiime2 diversity plug-in was used to analyze the alpha and beta diversity of the formed OTU representative sequences. The Kruskal–Wallis test and LEfSe analysis were used to analyze the significant differences between species at various classification levels.

### Detection of serum SCFAs by gas chromatography (GC–MS)

The researchers used serum samples to identify SCFAs through gas chromatography-mass spectrometry (GC–MS). They employed an Agilent GC–MS/MS (7890B-7000D) gas chromatography-mass spectrometer, known for its sensitivity and accuracy, to carry out the analysis.

### RNA extraction and qPCR

Total RNA was extracted from the KGN cells and mice ovary using the TRIzol method (Invitrogen, USA) as described in the product manual. The extracted RNA was quantified using NanoDrop (Thermo Scientific, USA). The total RNA was reverse transcribed according to the instructions on the reverse transcription kit HiScript III 1st Strand cDNA Synthesis Kit (Vazyme, China). The total RNA was reverse transcribed on ice and stored at − 80 °C. The primer was synthesized by Qing Ke Biotechnology Service Company (Additional file 2: Table S1). The cDNA obtained through reverse transcription was used as the template for the qRT-PCR reaction according to SYBR qPCR Master Mix instructions (Vazyme, China) of cDNA synthesis. After the reaction, the amplification and melting curves of Real-time PCR were confirmed, and the data were analyzed using the 2^−ΔΔCT^ method.

### Design and interference efficiency measurement of siRNA targeting FOSL2

Design and synthesize siRNA targeting FOSL2, cultivate KGN cells to a transfectable state and transfect with siRNA according to the transfection instructions. After 48 h of cultivation, extract RNA and protein from KGN cells in advance and examine the mRNA and protein expression levels of FOSL2, the sequence of siRNA is provided in Additional file 2: Table S1.

### H&E staining and follicle counting

Ovarian tissues were fixed with 4% paraformaldehyde (PFA) (Beyotime, China) at room temperature for 24 h and embedded in paraffin. Subsequently, the ovarian tissues were cut into 4 μm thick sections, dewaxed, hydrated, and stained with hematoxylin for 5–10 min. The ovarian histomorphological changes of mice in each group were observed under the microscope.

### Immunofluorescence

The cell creeper was put into a 6-well plate. When the fusion rate of KGN cells on the creeper reached 70%—80%, the cell creeper was fixed with 4% PFA for 15 min. Subsequently, the cells were permeated in 0.5% Triton X-100 for 20 min, washed thrice with phosphate buffer (PBS), and incubated with BSA at room temperature for 30 min. The cells were hybridized overnight at 4 °C with primary antibody to the anti-GLUT4 antibody (1:100) (Proteintch, China). The fluorescent-labeled secondary antibody was incubated at room temperature for 2 h and washed thrice using PBS. Then, DAPI was incubated at room temperature in the dark for 1 min and observed under a fluorescent microscope.

### Western blot

The total protein sample concentration was detected using the BCA method. In this study, 40 μg of each total protein sample was used for Western blotting. Proteins separated on the gel were transferred to a polyvinylidene fluoride (PVDF) membrane (250 mA, 80 min) and the membrane incubated in 5% skim milk powder at room temperature for one hour. The membranes were incubated overnight at 4 °C with PPAR-γ, GLUT4, METTL3, FOSL2, IL-6, TNF-α, NLRP3 antibodies. The next day, the membrane was washed three times with TBST and incubated with anti-horseradish peroxidase (HRP)-labeled sheep anti-rabbit IgG (1:5000) (Fudebio, China) for 2 h at room temperature. After incubation, the membrane was washed three times using TBST. The grayscale values of the protein bands were detected using chemiluminescence. The relative expression of the detected protein was expressed as the ratio of the grayscale values of the target protein to the Tubulin bands.

### Cell culture

The human ovarian granulosa cell line, designated KGN, was cultured in DMEM/F12 medium (Invitrogen, USA) containing 10% FBS (Invitrogen, USA) and 1% penicillin/streptomycin (Invitrogen, USA) and incubated at 37 °C with 5% CO_2_. The 100ug/ml of LPS in DMEM/F12 media was used in KGN cells for 24 h in 6-well cell plates. The granulosa cells were treated with several doses of butyric acid (BA) (1.1 mg/ml and 11 mg/ml) in DMEM/F12 media for 24 h in 6-well cell plates.

### Glucose uptake assay

The ability of KGN cells to uptake glucose was evaluated by measuring the fluorescent glucose 2-NBDG (APEXBIO, USA). The KGN cells seeded in 96-well plates were cultured in DMEM/F12 medium. The cells were gently rinsed with DPBS and incubated with 10 μM 2-NBDG at 37 °C for 10 min. The fluorescent intensity of the cells was measured using fluorescence microscopy (Olympus, Japan) with a 450 nm excitation. The level of intracellular fluorescence was quantitatively analyzed using ImageJ software.

### Mitochondrial membrane potential measurement

The mitochondrial membrane potential of KGN cells was measured using fluorescent probe JC-1 (Beyotime, China). The cells were incubated with JC-1 (10 μm) at 37 °C for 20 min in the dark. Subsequently, the cells were rinsed with DPBS twice. Fluorescent images of the cells were taken under a fluorescent microscope (Olympus, Japan). When the mitochondrial membrane potential is high, JC-1 aggregates in the mitochondrial matrix, forming a polymer that produces red fluorescence. When the mitochondrial membrane potential is low, JC-1 cannot accumulate in the mitochondrial matrix and exists as a monomer, resulting in green fluorescence. The level of intracellular fluorescence was quantitatively analyzed using ImageJ software. The red-to-green fluorescence ratio is often used to compare the mitochondrial membrane potential between each group of cells.

### RNA m6A quantification

Total RNA was isolated using the TRIzol reagent (Invitrogen, USA), and 100 ng of the total RNA was used for RNA m6A quantification. The m6A methylation levels in total RNA were evaluated using the EpiQuik m6A RNA Methylation Quantification Kit (Epigentek, USA). A standard curve was constructed according to the manufacturer's instructions. The RNA samples were coated on the strip wells and incubated with capture antibodies. After incubation, the strip wells were washed with washing buffer, and detection antibody and enhanced solution were added. The detection solution created the signals for RNA m6 quantification. The m6A content was quantified by reading the absorbance of samples at 450 nm and calculated according to the standard curve.

### Methylated RNA immunoprecipitation sequencing (MeRIP-seq) and MeRIP-qPCR

The m6A RNA high-throughput sequencing technology service was provided by Hangzhou Lianchuang Biotechnology Co., Ltd. Methylated RNA Immunoprecipitation (MeRIP) was performed using EpiQuik MeRIP m6A Kit (Epogentek, USA) following the manufacturer's instructions. To perform MeRIP, total RNA was extracted using Trizol reagent (Invitrogen, CA, USA). Subsequently, Agilent 2100 bioanalyzer (Agilent, CA, USA) was used to analyze the quality and quantity of total RNA. The Epicentre Ribo-Zero Gold Kit (Illumina, San Diego, USA) was used to purify RNA and remove rRNA. On the other hand, m6A antibody (Synaptic Systems, Germany) was used to bind related RNA fragments, and enrich them with protein-A beans. The eluted fragment containing m6A (IP) and the untreated input control fragment were converted into the final cDNA library. Finally, sequencing was performed using an Illumina Novaseq™ 6000 platform according to the manufacturer’s instructions.

Real-time quantitative RT-PCR analysis (qRT-PCR) was performed using samples with anti-m6A antibody, normal IgG, and input samples in triplicates. The threshold cycle (CT) value of the samples with anti-m6A antibodies were normalized to input by subtracting the Ct of input from the Ct of IP samples: ∆Ct = Ct_IP_ − (Ct_input_ − Log_2_[Input Dilution Factor]). Then, the percent of input for each IP sample was calculated: % Input = 2 − ΔCt _(normalized IP)._ The primers for MeRIP-qPCR are listed in Additional file 2: Table S1.

### Mice

Specific-pathogen-free (SPF) grade C57/B6 mice (6–8 weeks old) were purchased from Weitong Lihua Co. (Beijing, China). The mice were housed at constant temperature and humidity, and 12 h light/dark cycle a day and provided with adequate food and water. The experimental animal ethics committee of the Affiliated Hospital of Nanjing University of Chinese Medicine approved the animal experiment.

### Measurement of serum concentrations by ELISA

The mice were anesthetized, and blood was drawn from the abdominal aorta and allowed to stand for 2 h. Subsequently, the blood was centrifuged to obtain serum. The serum sex hormone indexes and concentrations of FSH, LH, E2, T, and insulin were determined using ELISA kits purchased from Bioswamp (Wuhan, China). The ELISA test was performed by diluting the samples to 100μL (1:20) and incubating them with specific capture and detection antibodies according to the manufacturer’s instructions. All samples were detected at 450 nm optical density.

### Statistical analyses

All data analyses were conducted using SPSS 20.0 statistical software. All data were expressed as mean ± standard deviation (SD). One-way ANOVA was used to determine the significant difference. A *P* value of less than 0.05 was considered significant. After finding significant differences, a post hoc analysis was performed using a Tukey honest significant difference test. The Kruskal–Wallis test was used for not normally distributed values. The Mothur software was used to analyze α Diversity and visually display species annotation results. On the other hand, β-Diversity analysis was calculated using Qiime software (v1.7.0) to evaluate differences in the species complexity between samples. The LEfSe difference analysis of species was performed to find the communities or species that significantly impact the differences between groups. The results demonstrated that the LDA threshold was 2.0.

## Supplementary Information

**Additional file 1: Fig. S1**. Flow chart showing patients recruitment. **Fig. S2**. A Composition of species at genus level among three groups. B Sequence logo representing consensus motif of m6A sites in two groups granulosa cell peaks by MeRIP-seq. C After transfection with siRNA-FOSL2 in three groups of KGN cells, the mRNA expression levels of FOSL in the cells were measured. *P < 0.05. D, E After transfection with siRNA-FOSL in three groups of KGN cells, the protein expression levels of FOSL2 in the cells were measured. *P < 0.05. F, G Knockdown of FOSL2 in KGN cells and the protein expression levels of IL-6 and TNF-α in the cells. *P < 0.05. H Body weight of mice in each group. Bars represent means ± SD, n = 5. *P < 0.05

**Additional file 2: Table S1**. PCR primers.

## Data Availability

The datasets presented in this study can be found in online repositories. The name of the repository and accession number can be found below. National Center for Biotechnology Information; PRJNA934844 and PRJNA934689. (https://www.ncbi.nlm.nih.gov/bioproject/PRJNA934844, https://www.ncbi.nlm.nih.gov/bioproject/PRJNA934689).

## Abbreviations

PCOS: Polycystic ovary syndrome
IVF: In vitro fertilization
M6A: N6-methyladenosine
IL-6: Interleukin-6
TNF-α: Tumor necrosis factor-α
TGF-β1: Transforming Growth Factor Beta-1
LPS: Lipopolysaccharide
Me-RIP: Methylated RNA immunoprecipitation sequencing
BMI: Body mass index
AMH: Anti-Müllerian hormone
FSH: Follicle-stimulating hormone
LH: Luteinizing hormone
T: Testosterone
OTU: Operational Taxonomic Unit
KGN: Human ovarian granulosa tumor
GC-MS: Gas chromatography-mass spectrometry
SCFAs: Short-chain fatty acids
rRNA: Ribosomal ribonucleic acid
