# Supplementary material for: Short-chain fatty acid-butyric acid ameliorates granulosa cells inflammation through regulating METTL3-mediated N6-methyladenosine modification of FOSL2 in polycystic ovarian syndrome
Source: Clin Epigenetics. 2023 May 13;15:86. doi: 10.1186/s13148-023-01487-9 (PMC10183145; doi:10.1186/s13148-023-01487-9)
Supplement: Supplementary file 1 — Additional file 1: Fig. S1. Flow chart showing patients recruitment. Fig. S2. A Composition of species at genus level among three groups. B Sequence logo representing consensus motif of m6A sites in two groups granulosa cell peaks by MeRIP-seq. C After transfection with siRNA-FOSL2 in three groups of KGN cells, the mRNA expression levels of FOSL in the cells were measured. *P < 0.05. D, E After transfection with siRNA-FOSL in three groups of KGN cells, the protein expression levels of FOSL2 in the cells were measured. *P < 0.05. F, G Knockdown of FOSL2 in KGN cells and the protein expression levels of IL-6 and TNF-α in the cells. *P < 0.05. H Body weight of mice in each group. Bars represent means ± SD, n = 5. *P < 0.05 [file 13148_2023_1487_MOESM1_ESM.pptx]

## Slide 1
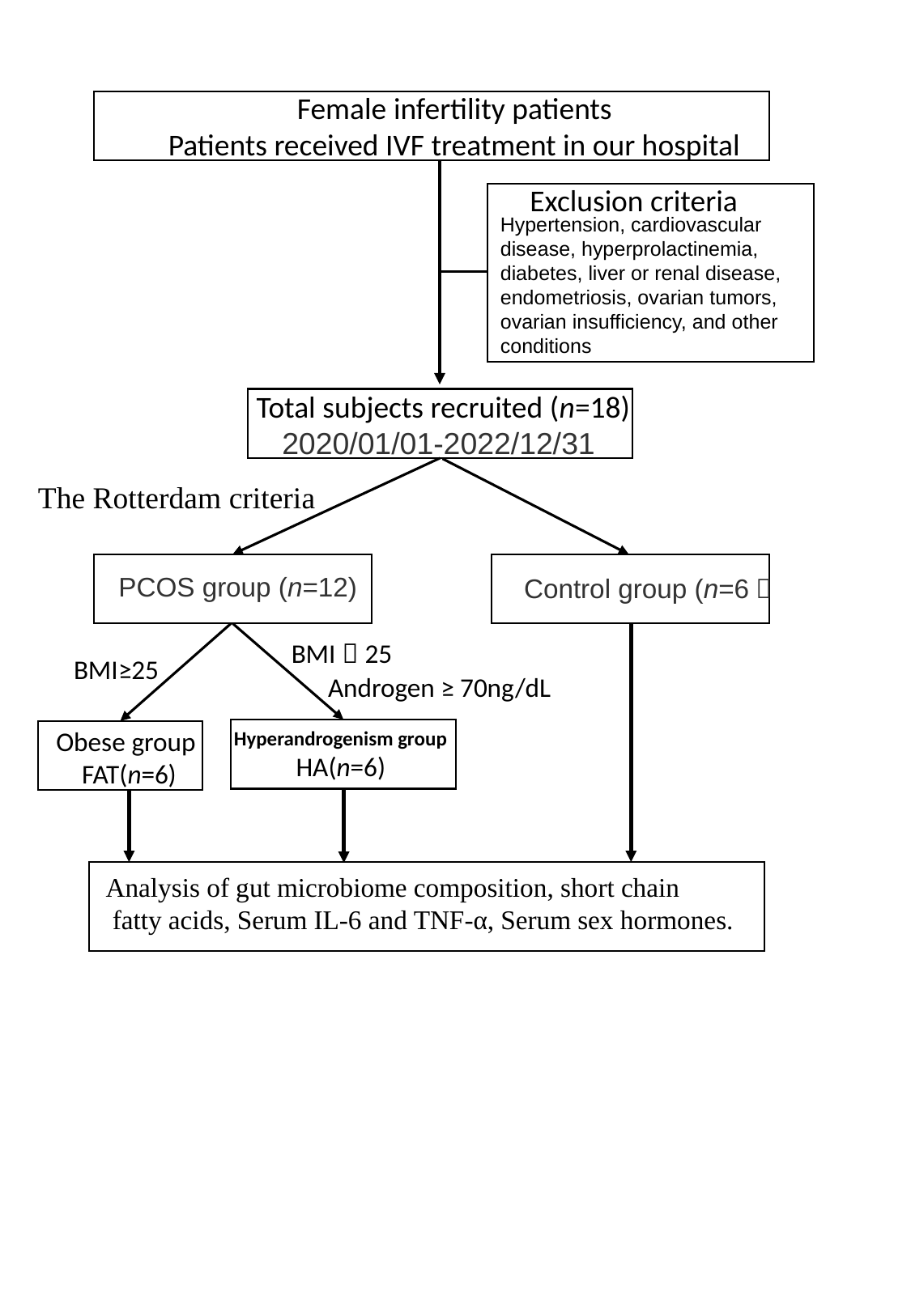

Female infertility patients
Patients received IVF treatment in our hospital
Total subjects recruited (n=18)
 2020/01/01-2022/12/31patients
The Rotterdam criteria
PCOS group (n=12)
Control group (n=6）
BMI＜25
BMI≥25
Obese group
FAT(n=6)
Hyperandrogenism group
HA(n=6)
Analysis of gut microbiome composition, short chain
 fatty acids, Serum IL-6 and TNF-α, Serum sex hormones.
Exclusion criteria
Hypertension, cardiovascular disease, hyperprolactinemia, diabetes, liver or renal disease, endometriosis, ovarian tumors, ovarian insufficiency, and other conditions
Androgen ≥ 70ng/dL

## Slide 2
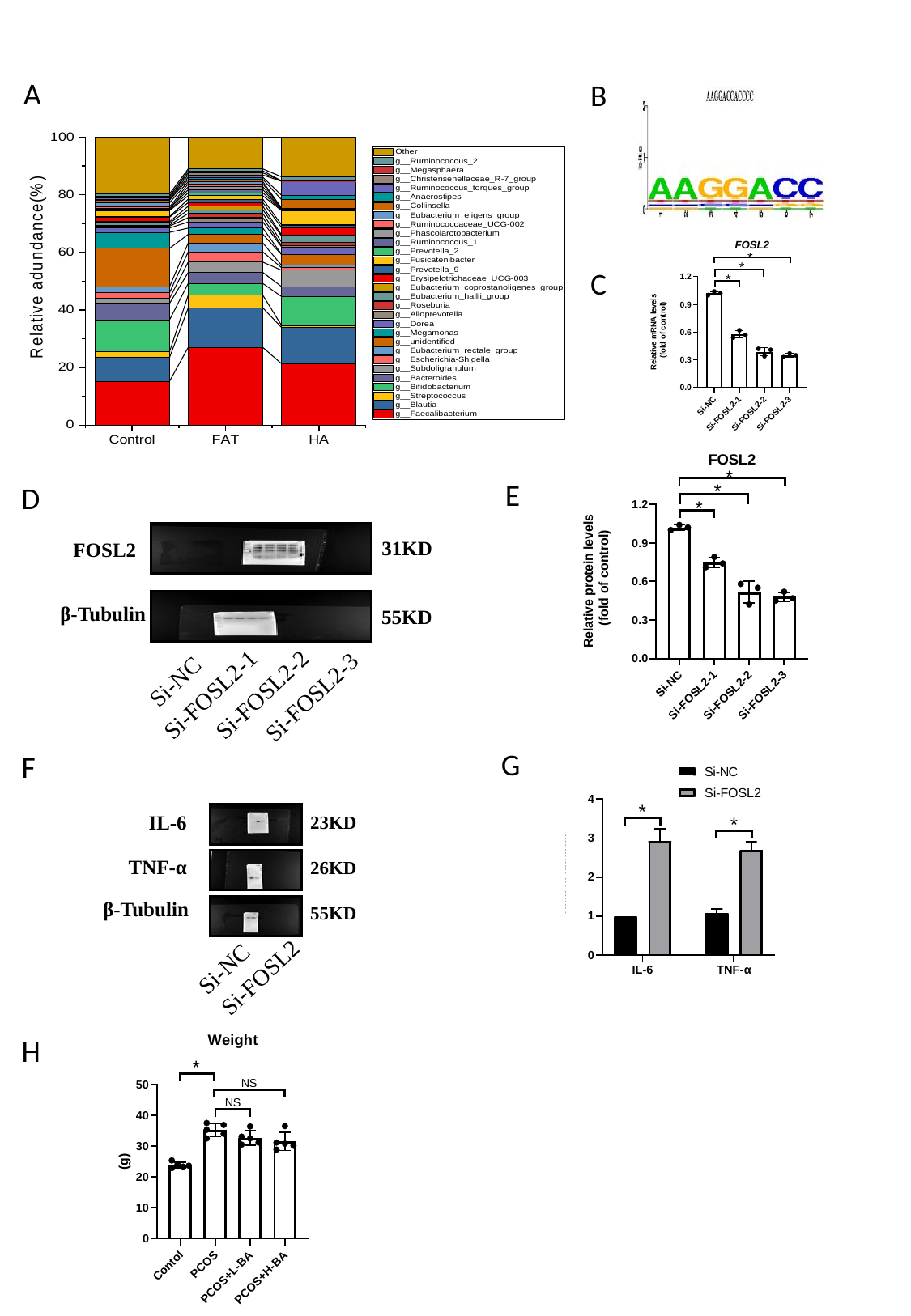

A
B
C
E
D
31KD
FOSL2
β-Tubulin
55KD
Si-NC
Si-FOSL2-1
Si-FOSL2-2
Si-FOSL2-3
G
F
IL-6
23KD
TNF-α
26KD
β-Tubulin
55KD
Si-NC
Si-FOSL2
H
